# Gastroesophageal reflux disease and risk of cancer: Findings from the Korean National Health Screening Cohort

**DOI:** 10.1002/cam4.6500

**Published:** 2023-09-07

**Authors:** Chi Lan Tran, Minji Han, Byungmi Kim, Eun Young Park, Young Il Kim, Jin‐Kyoung Oh

**Affiliations:** ^1^ Department of Cancer Control and Population Health Graduate School of Cancer Science and Policy, National Cancer Center Goyang‐si Gyeonggi‐do Republic of Korea; ^2^ Division of Cancer Prevention National Cancer Center Goyang‐si Gyeonggi‐do Republic of Korea; ^3^ Department of Health Science and Technology, Graduate School of Convergence Science and Technology Seoul National University Seoul Republic of Korea; ^4^ Department of Preventive Medicine Korea University College of Medicine Seoul Republic of Korea; ^5^ Center for Gastric Cancer National Cancer Center Goyang‐si Gyeonggi‐do Republic of Korea

**Keywords:** cancer risk, cohort, gastroesophageal reflux disease, laryngeal cancer

## Abstract

**Aim:**

Little is known about the association of cancers other than esophageal adenocarcinoma with gastroesophageal reflux disease (GERD). This study aimed to examine the association between GERD and the risk of different types of cancer.

**Methods:**

A cohort study was conducted using data from the National Health Screening Cohort. We included 10,261 GERD patients and 30,783 non‐GERD individuals who were matched in a 1:3 ratio by age and sex. All participants were followed‐up until cancer diagnosis, death, or end of the study (December 31, 2015). Hazard ratios were calculated using the Cox proportional hazards model, adjusting for smoking and alcohol consumption, physical activity, body mass index, income, area, and Charlson Comorbidity Index.

**Results:**

The median follow‐up time was 9.9 years. GERD was associated with an increased risk of esophageal (adjusted hazard ratios [aHR] = 3.20 [1.89–5.41]), laryngeal (aHR = 5.42 [2.68–10.96]), and thyroid cancers (aHR = 1.91 [1.55–2.34]) after controlling for all covariates. The results were consistent when examining GERD with esophagitis (K210) and without esophagitis (K219) separately. For thyroid cancer, the results were insignificant after controlling for having ever‐received thyroid biopsy procedures. A dose–response relationship was found between GERD and esophageal cancer as well as laryngeal cancer, with patients with a longer duration of GERD treatment showing a stronger effect. In contrast, GERD was associated with a reduced risk of colorectal (aHR = 0.73 [0.59–0.90]), liver (aHR = 0.67 [0.51–0.89]), and pancreatic cancers (aHR = 0.43 [0.24–0.76]), which might have resulted from differences in healthcare utilization between GERD and non‐GERD groups.

**Conclusion:**

GERD was associated with an increased risk of esophageal and laryngeal cancers. Additionally, early detection and treatment of precancerous lesions among the GERD group could lead to a lower risk of colorectal, liver, and pancreatic cancers.

## 1 INTRODUCTION

Gastroesophageal reflux disease (GERD) is a common digestive condition caused by the backflow of stomach contents into the esophagus or oral cavity, resulting in symptoms and complications such as heartburn and regurgitation.^1^, ^2^ GERD is associated with esophageal obstruction, erosive esophagitis, Barrett's esophagus, and esophageal adenocarcinoma.^3^ Recent evidence suggests that GERD affects billions of people worldwide, with its burden continuing to grow as a result of aging and population growth.^4^, ^5^, ^6^

GERD can cause chronic inflammation that can contribute to cancer development. The relationship between GERD and cancer has been well‐established for esophageal adenocarcinoma.^7^ This association may be due to repeated exposure to stomach acid, leading to inflammation, damage, and changes in the esophagus epithelium, causing Barrett's esophagus and eventually esophageal cancer.^8^ The stomach acid can also reach the larynx, pharynx, oral cavity, and lung, damaging these organs and leading to cancer. Several recent publications support the positive association of GERD with cancer at those sites, especially for laryngeal cancer, with the significant results from two recent meta‐analyses of case–control studies.^9^, ^10^ However, case–control studies cannot rule out the possibility of reverse causation. For other head and neck cancers, the results are still controversial. While the meta‐analysis of 13 case–control studies found no association with GERD,^9^ other studies have showed that GERD increases the risk of malignancy in the upper aerodigestive tract.^11^, ^12^ The association between GERD and lung cancer remains unknown. However, the evidence suggests that GERD patients have a higher risk of lung diseases.^7^ Several cohort studies were also conducted. A Norwegian cohort study suggested that people with severe reflux symptoms have increased in esophageal adenocarcinoma‐specific mortality, although the absolute risk is small.^13^ A Taiwan cohort study found a significant association between GERD and CRC risk in both genders.^14^ Another cohort study in Taiwan also found that GERD may increase the risk of lung cancer.^15^ A recent cohort study in Korea also found significant increased risk of larynx cancer among GERD individuals.^16^

Nevertheless, in most studies, possible confounders such as smoking status, alcohol consumption, physical activities, body mass index (BMI), and comorbidities were not considered owing to a lack of information. Other than esophageal adenocarcinoma, there is little evidence demonstrating a causal relationship between GERD and other cancers, with the available results being controversial. Therefore, in this study, we evaluated the relationship between GERD and the risk of different types of cancer using a matched cohort study design while considering the possible confounders.

## 2 METHODS

### 2.1 Data source

We used data from the National Health Insurance Service‐National Health Screening Cohort (NHIS‐HEALS). NHIS is a single mandatory national health insurance that covers Korea's entire population and provides a general health screening program to all beneficiaries every 1 or 2 years. NHIS‐HEALS is a randomly selected cohort comprised 10% of the screening participants during 2002–2003 who were followed‐up until 2015.^17^ The cohort includes 514,866 people aged 40–79 years with essential information, including sociodemographic factors, self‐reported health behaviors, clinical laboratory results, and healthcare usage based on insurance claim data.^17^

### 2.2 Assessment of exposure

**FIGURE 1:**
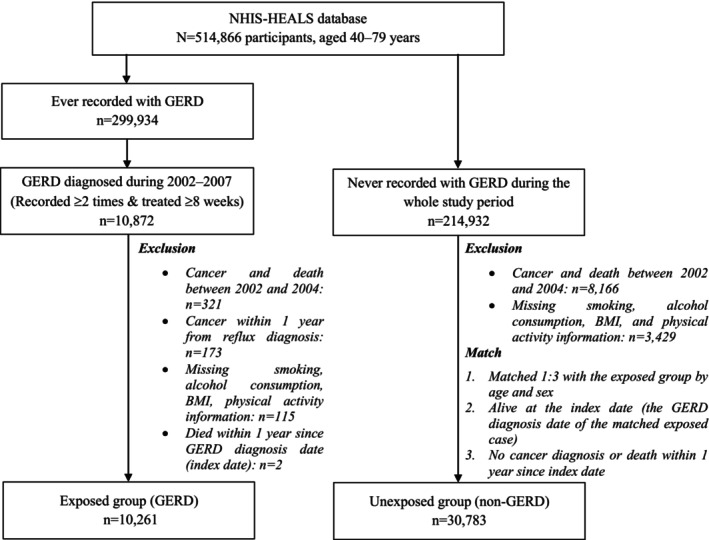
Flowchart of study. GERD, gastroesophageal reflux disease.

Individuals with the International Classification of Diseases, 10th Revision (ICD‐10) code “K210” (GERD with esophagitis) or “K219” (GERD without esophagitis) concurrent with a prescription of proton pump inhibitor (PPI) or histamine‐2 receptor antagonists (H2RA) were considered as “ever recorded with GERD” (Figure 1). Among NHIS‐HEALS participants, 299,934 were recorded with GERD during 2002–2015. To ensure the accuracy of GERD diagnosis, we included individuals recorded with GERD at least twice and treated for GERD with PPI or H2RA for at least 8 weeks as GERD patients. These criteria were based on guidelines for the treatment of GERD in Korea, which recommend the initial treatment of standard dose PPI (or H2RA as an alternative agent) for at least 4–8 weeks, with continuous treatment required for most GERD patients afterward.^18^, ^19^

### 2.3 Study subjects

To create the exposed group (GERD), we identified 10,872 participants who satisfied the criteria for GERD diagnosis during 2002–2007. Then, we excluded participants who died or were diagnosed with cancer between 2002 and 2004 to create a cancer‐free cohort (*n* = 321). We also excluded participants who had cancer before or within 1 year after GERD diagnosis to ensure that cancer did not present before developing GERD (*n* = 173). Participants with missing information on lifestyle risk factors and who died within 1 year of GERD diagnosis (index date) were also excluded. Finally, 10,261 patients were included in the exposed group (Figure 1).

To create the unexposed group (non‐GERD), we identified 214,932 participants who had never been recorded with GERD during the whole study period. We excluded participants who were dead or diagnosed with cancer during 2002–2004 and those with missing lifestyle risk factor information. We then matched one exposed individual with three unexposed individuals by age and sex, using individual matching. The index date of the unexposed individual was defined as the GERD diagnosis date of the matched exposed individual. Unexposed individuals must be alive at the index date and without cancer diagnosis or death within 1 year since the index date. Finally, 30,783 individuals were included in the unexposed group (Figure 1).

Cancer diagnoses were observed from 1 year after the index date until December 31, 2015. The lag time of 1 year was applied for both groups to prevent protopathic bias and ensure a similar follow‐up period between the two groups.

### 2.4 Assessment of outcomes

Cancer patients were defined as being recorded with a cancer ICD‐10 code at least once as an inpatient or three times as an outpatient between 2002 and 2015. Specific cancer outcomes include esophageal cancer (C15), stomach cancer (C16), colorectal cancer (C18, C19, C20), liver cancer (C22), pancreatic cancer (C25), laryngeal cancer (C32), lung cancer (C33‐C34), and thyroid cancer (C73). Other specific cancers were not included due to a small number of cancer cases or lack of information.

### 2.5 Covariates

Lifestyle risk factors were collected by structured questionnaires during general health screening during 2002–2007. Smoking was classified as none, former, current smoking <20 pack‐years, and current smoking ≥20 pack‐years based on smoking history. Alcohol consumption was classified into three groups: none, light drink, and heavy drink (more than two standard drinks per day for men and more than one standard drink per day for women, where one standard drink equals 14 g of alcohol).^20^ Participants who answered “do not exercise” were considered physically inactive. BMI was measured directly using body weight and height, classified into three groups: <23, 23 to <25, and ≥25. Age, sex, income, and residential area information were recorded in the baseline year 2002. Income was identified using the NHIS premiums, with 10 quintiles corresponding to participants' household income, from lowest (1st) to highest (10th), and 0th quintile representing the Medicaid population (lowest income group). Income was reclassified into three groups as low (0th–3rd quintiles), middle (4th–7th quintiles), and high (8th–10th quintiles). The residential area was divided by the metropolitan city (Seoul, Busan, Daegu, Incheon, Gwangju, Daejeon, and Ulsan) and provinces (other areas). The Charlson Comorbidity Index (CCI) was calculated using data obtained between 2002 and 2007. Chronic viral hepatitis B or C was identified using ICD‐10 code B18.

### 2.6 Statistical analysis

Characteristics of the GERD and non‐GERD groups were described using descriptive analysis. We compared the two groups using the chi‐squared test for categorical variables and the two‐sample *t*‐test for continuous variable (alpha error = 0.05, two‐sided testing).

Hazard ratios (HRs) and 95% confidence intervals (CIs) were calculated using the Cox proportional hazard (Cox PH) model to examine the risk of cancer in the GERD group compared to that in the non‐GERD group. *p* < 0.05 was considered as the significant threshold. Person‐year was calculated from the index date until cancer diagnosis, death, or end of study (December 31, 2015). Two models were constructed: Model 1 adjusted for age and sex and Model 2 adjusted for age, sex, smoking status, alcohol consumption, physical activity, BMI, income, area, and CCI. For liver cancer, we additionally adjusted for chronic viral hepatitis B or C. We also conducted analyses separately for GERD with esophagitis (K210) and GERD without esophagitis (K219).

We investigated the impact of GERD on cancer risk according to the duration of GERD treatment. We calculated the cumulative duration of PPI/H2RA treatment and cumulative duration of PPI‐only treatment for GERD of each participant during the whole study period. Long treatment duration was defined as ≥360 or ≥540 cumulative days. HRs were calculated using the Cox PH model, with the non‐GERD group as the reference group. The model was adjusted for age, sex, smoking status, alcohol consumption, physical activity, BMI, income, residential area, and CCI. The *p* for linear trend was calculated by modeling the variable as a continuous variable.

Additional analyses were conducted to calculate and compare the number of health checkups and number of hospital visits per year during the whole study period between the GERD and non‐GERD groups. These two variables were then added to the Cox PH model to observe the effect of GERD on cancer outcomes after adjusting for differences in healthcare utilization between the two groups. We also examined the proportion of receiving thyroid biopsy procedures (needle biopsy or operation biopsy) and polypectomy procedures between the two groups. The variable of receiving a thyroid biopsy procedure was added to the Cox PH model to observe the effect of GERD on thyroid cancer. All data analyses were performed using SAS Enterprise Guide Software (version 7.1, SAS Institute).

## 3 RESULTS

### 3.1 Characteristics of study subjects

In total, 10,261 and 30,783 participants with and without GERD were included, with a median follow‐up time of 9.9 years. Age and sex were matched by the individual matching procedure. The mean age of the study population was 54.4 years, and 50.92% of participants were male. Other covariates significantly differed between the two groups. In general, the GERD group had a slightly lower percentage of current smokers, heavy drinkers, and physically inactive individuals. Contrastingly, the GERD group had a higher proportion of individuals with a BMI ≥25, chronic viral hepatitis, and CCI ≥1. For sociodemographic factors, the GERD group had a higher proportion of high‐income participants and people living in metropolitan cities (Table 1).

**TABLE 1:** Characteristics of the study population.

Characteristics	GERD group (*N* = 10,261)	Non‐GERD group (*N* = 30,783)	*p*‐value
	*n* (%)	*n* (%)	
Sex			Matched
Male	5225 (50.92)	15,675 (50.92)	
Female	5036 (49.08)	15,108 (49.08)	
Age			Matched
Mean (SD)	54.40 (8.80)	54.40 (8.80)	
40–49	3455 (33.67)	10,365 (33.67)	
50–59	3702 (36.08)	11,106 (36.08)	
60–69	2607 (25.41)	7821 (25.41)	
70–79	497 (4.84)	1491 (4.84)	
Smoking			<0.0001^a^
None	6541 (63.75)	19,524 (63.42)	
Former	1218 (11.87)	3055 (9.92)	
Current, <20 pack‐years	1163 (11.33)	4056 (13.18)	
Current, ≥20 pack‐years	1339 (13.05)	4148 (13.48)	
Alcohol consumption			0.0034^a^
None	5431 (52.93)	15,815 (51.38)	
Light drink	872 (8.50)	2897 (9.41)	
Heavy drink	3958 (38.57)	12,071 (39.21)	
Physical activity			<0.0001^a^
Physically inactive	3463 (33.75)	11,531 (37.46)	
BMI			<0.0001^b^
Mean (SD)	24.83 (2.93)	24.66 (3.09)	
BMI (<23)	2717 (26.48)	9159 (29.75)	
BMI (23 – <25)	2711 (26.42)	8173 (26.55)	
BMI (≥25)	4833 (47.10)	13,451 (43.70)	
Viral Hepatitis B or C	289 (2.82)	455 (1.48)	<0.0001^a^
CCI			<0.0001^a^
CCI ≥1	1502 (14.64)	3780 (12.28)	
Income			<0.0001^a^
Low (0th–3rd)	2039 (19.87)	7804 (25.35)	
Middle (4th–7th)	3225 (31.43)	10,124 (32.89)	
High (8th–10th)	4997 (48.70)	12,855 (41.76)	
Area			0.0208^a^
Metropolitan city	4760 (46.39)	13,876 (45.08)	
Province	5501 (53.61)	16,907 (54.92)	

Abbreviations: CCI, Charlson Comorbidity Index; GERD, gastroesophageal reflux disease; SD, standard deviation.

^a^ Chi‐squared test.

^b^ Two‐sample *t*‐test.

### 3.2 Hazard ratios of GERD for cancer

We identified 1032 and 3060 cancer cases in the GERD and non‐GERD groups. Overall, GERD was associated with an increased risk of esophageal cancer (adjusted hazard ratios [aHR] = 3.20 [1.89–5.41]), laryngeal cancer (aHR = 5.42 [2.68–10.96]), and thyroid cancer (aHR = 1.91 [1.55–2.34]) after controlling for age, sex, smoking status, alcohol consumption, physical activity, BMI, income, area, and CCI. In contrast, GERD was associated with a decreased risk of colorectal (aHR = 0.73 [0.59–0.90]), liver (aHR = 0.67 [0.51–0.89]), and pancreatic cancers (aHR = 0.43 [0.24–0.76]). No significant result was found for stomach and lung cancer (Table 2).

**TABLE 2:** Hazard ratios of gastroesophageal reflux disease for cancer.

Outcomes	Cancer cases		Model 1^a^			Model 2^b^		
	GERD	Non‐GERD	HR	95% CI	*p*‐value	HR	95% CI	*p*‐value
	(*N* = 10,261)	(*N* = 30,783)						
Person‐years	103,906	298,231						
All cancer	1032	3060	0.97	(0.90–1.04)	0.324	1.03	(0.96–1.10)	0.461
Esophageal cancer	28	31	2.59	(1.55–4.31)	0.0003	3.2	(1.89–5.41)	<0.0001
Stomach cancer	165	511	0.93	(0.78–1.10)	0.384	0.99	(0.83–1.19)	0.944
Colorectal cancer	105	437	0.69	(0.56–0.85)	0.001	0.73	(0.59–0.90)	0.004
Liver cancer	61	295	0.59	(0.45–0.78)	0.0002	0.67	(0.51–0.89)	0.005
Pancreatic cancer	13	99	0.38	(0.21–0.67)	0.001	0.43	(0.24–0.76)	0.004
Laryngeal cancer	22	13	4.84	(2.44–9.61)	<0.0001	5.42	(2.68–10.96)	<0.0001
Lung cancer	107	381	0.8	(0.65–1.00)	0.05	0.92	(0.74–1.14)	0.445
Thyroid cancer	152	226	1.92	(1.57–2.36)	<0.0001	1.91	(1.55–2.34)	<0.0001

Abbreviations: GERD, gastroesophageal reflux disease; PYs, person‐years; HR, hazard ratio; CI, confidence interval.

^a^ Model 1 adjusted for age and sex.

^b^ Model 2 adjusted for all covariates, including age, sex, smoking status, alcohol consumption, physical activity, BMI, income, area, and Charlson Comorbidity Index; liver cancer was additionally adjusted for chronic viral hepatitis B or C.

### 3.3 Subgroup analyses by GERD with esophagitis and GERD without esophagitis

For GERD with esophagitis, a higher risk of esophageal (aHR = 3.19 [1.77–5.74]), laryngeal (aHR = 6.24 [2.64–14.71]), and thyroid cancers (aHR = 1.99 [1.57–2.52]) and a lower risk of colorectal, liver, and pancreatic cancers were found among the GERD group after controlling for all covariates (Table 3).

**TABLE 3:** Hazard ratios for cancer by gastroesophageal reflux disease subtypes.

Outcomes	Cancer cases		Model 1^a^			Model 2^b^		
	GERD	Non‐GERD	HR	95% CI	*p*‐value	HR	95% CI	*p*‐value
**GERD with esophagitis (K210)**								
No. of participants	8104	24,312						
Person‐years	82,641	237,168						
All cancer	823	2414	0.98	(0.90–1.06)	0.533	1.05	(0.97–1.14)	0.223
Esophageal cancer	22	25	2.52	(1.42–4.46)	0.002	3.19	(1.77–5.74)	0.0001
Stomach cancer	129	413	0.89	(0.73–1.09)	0.268	0.96	(0.78–1.17)	0.672
Colorectal cancer	84	346	0.69	(0.55–0.88)	0.003	0.74	(0.59–0.95)	0.016
Liver cancer	55	231	0.68	(0.51–0.92)	0.011	0.72	(0.54–0.98)	0.035
Pancreatic cancer	10	78	0.37	(0.19–0.71)	0.003	0.43	(0.22–0.83)	0.012
Laryngeal cancer	16	9	5.11	(2.26–11.56)	<0.0001	6.24	(2.64–14.71)	<0.0001
Lung cancer	89	319	0.8	(0.63–1.01)	0.06	0.93	(0.73–1.18)	0.543
Thyroid cancer	119	172	1.98	(1.57–2.50)	<0.0001	1.99	(1.57–2.52)	<0.0001
**GERD without esophagitis (K219)**								
No. of participants	2157	6471						
Person‐years	21,265	61,063						
All cancer	209	646	0.93	(0.79–1.08)	0.339	0.94	(0.81–1.11)	0.472
Esophageal cancer	6	6	2.89	(0.93–8.95)	0.067	3.35	(1.03–10.88)	0.045
Stomach cancer	36	98	1.06	(0.72–1.55)	0.784	1.12	(0.76–1.64)	0.573
Colorectal cancer	21	91	0.66	(0.41–1.07)	0.09	0.68	(0.42–1.09)	0.107
Liver cancer	6	64	0.27	(0.12–0.62)	0.002	0.28	(0.12–0.65)	0.003
Pancreatic cancer	3	21	0.41	(0.12–1.37)	0.149	0.45	(0.13–1.52)	0.201
Laryngeal cancer	6	4	4.23	(1.19–14.99)	0.026	4.57	(1.27–16.45)	0.02
Lung cancer	18	62	0.83	(0.49–1.40)	0.487	0.87	(0.51–1.47)	0.594
Thyroid cancer	33	54	1.75	(1.13–2.70)	0.012	1.66	(1.08–2.57)	0.022

Abbreviations: CI, confidence interval; GERD, gastroesophageal reflux disease; HR, hazard ratio; PYs, person‐years.

^a^ Model 1 adjusted for age and sex.

^b^ Model 2 adjusted for all covariates, including age, sex, smoking status, alcohol consumption, physical activity, BMI, income, area, and Charlson Comorbidity Index; liver cancer was additionally adjusted for chronic viral hepatitis B or C.

For GERD without esophagitis, a higher risk of esophageal (aHR = 3.35 [1.03–10.88]), laryngeal (aHR = 4.57 [1.27–16.45]), and thyroid cancers (aHR = 1.66 [1.08–2.57]) and a lower risk of liver cancer (aHR = 0.28 [0.12–0.65]) were found. On the other hand, no significant results were found for colorectal, pancreatic, stomach, and lung cancers (Table 3).

### 3.4 The effect of GERD on cancer according to the duration of GERD treatment

When further analyzed according to the duration of GERD treatment, the increase in the risk of esophageal and laryngeal cancers was significant in both short‐ and long‐duration GERD treatment groups compared to risk in the non‐GERD group. Besides, it was observed that the longer the duration of GERD treatment, the stronger the effect of GERD on esophageal and laryngeal cancers (Table 4). For esophageal cancer, GERD participants with <360 days of treatment showed 2.45 times higher risk (aHR = 2.45 [1.11–5.38]), while participants with ≥360 days of PPI/H2RA treatment showed 3.64 higher risk (aHR = 3.64 [2.05–6.47]) compared to the risk in the non‐GERD group. Similarly, for laryngeal cancer, aHRs and 95% CIs were 4.85 (1.90–12.41) and 5.72 (2.68–12.21) for GERD patients with <360 days and ≥360 days of treatment, respectively. The HRs for thyroid cancer also follow increasing trends corresponding to the longer duration of GERD treatment, with the *p* for trend being <0.0001. Consistent results were found when considering a duration cutoff of 540 days and when considering PPI treatment only. On the other hand, the decreasing trends were not clear and consistent for colorectal, liver, and pancreatic cancers (Table 4).

**TABLE 4:** Hazard ratios for cancer according to duration of GERD treatment.

		Case	HR^a^	95% CI		Case	HR^a^	95% CI
**PPI/H2RA treatment**								
Esophageal cancer	0 (non‐GERD)	31	Ref.		0 (non‐GERD)	31	Ref.	
	<360 days	8	2.45	(1.11–5.38)	<540 days	13	2.80	(1.45–5.42)
	≥360 days	20	3.64	(2.05–6.47)	≥540 days	15	3.65	(1.95–6.84)
	*p*‐trend		<0.0001		*p*‐trend		<0.0001	
Laryngeal cancer	0 (non‐GERD)	13	Ref.		0 (non‐GERD)	13	Ref.	
	<360 days	7	4.85	(1.90–12.41)	<540 days	12	5.85	(2.61–13.11)
	≥360 days	15	5.72	(2.68–12.21)	≥540 days	10	5.00	(2.16–11.55)
	*p*‐trend		<0.0001		*p*‐trend		<0.0001	
Thyroid cancer	0 (non‐GERD)	226	Ref.		0 (non‐GERD)	226	Ref.	
	<360 days	55	1.85	(1.37–2.48)	<540 days	86	2.02	(1.58–2.60)
	≥360 days	97	1.94	(1.53–2.46)	≥540 days	66	1.77	(1.34–2.33)
	*p*‐trend		<0.0001		*p*‐trend		<0.0001	
Colorectal cancer	0 (non‐GERD)	437	Ref.		0 (non‐GERD)	437	Ref.	
	<360 days	35	0.64	(0.46–0.91)	<540 days	50	0.65	(0.48–0.87)
	≥360 days	70	0.78	(0.61–1.01)	≥540 days	55	0.82	(0.62–1.09)
	*p*‐trend		0.014		*p*‐trend		0.023	
Liver cancer	0 (non‐GERD)	295	Ref.		0 (non‐GERD)	295	Ref.	
	<360 days	27	0.74	(0.50–1.09)	<540 days	40	0.78	(0.56–1.08)
	≥360 days	34	0.56	(0.39–0.80)	≥540 days	21	0.46	(0.29–0.72)
	*p*‐trend		0.0007		*p*‐trend		0.0003	
Pancreatic cancer	0 (non‐GERD)	99	Ref.		0 (non‐GERD)	99	Ref.	
	<360 days	5	0.44	(0.18–1.09)	<540 days	6	0.37	(0.16–0.85)
	≥360 days	8	0.42	(0.20–0.86)	≥540 days	7	0.49	(0.22–1.05)
	*p*‐trend		0.006		*p*‐trend		0.01	
**PPI treatment**								
Esophageal cancer	0 (non‐GERD)	31	Ref.		0 (non‐GERD)	31	Ref.	
	<360 days	10	2.42	(1.17–4.99)	<540 days	14	2.56	(1.35–4.87)
	≥360 days	18	3.90	(2.15–7.05)	≥540 days	14	4.27	(2.24–8.13)
	*p*‐trend		<0.0001		*p*‐trend			
Laryngeal cancer	0 (non‐GERD)	13	Ref.		0 (non‐GERD)	13	Ref.	
	<360 days	11	5.81	(2.56–13.2)	<540 days	15	5.98	(2.80–12.78)
	≥360 days	11	5.07	(2.23–11.5)	≥540 days	7	4.50	(1.17–11.47)
	*p*‐trend		<0.0001		*p*‐trend		<0.0001	
Thyroid cancer	0 (non‐GERD)	226	Ref.		0 (non‐GERD)	226	Ref.	
	<360 days	69	1.82	(1.39–2.38)	<540 days	98	1.97	(1.56–2.51)
	≥360 days	83	1.99	(1.54–2.56)	≥540 days	54	1.79	(1.33–2.41)
	*p*‐trend		<0.0001		*p*‐trend		<0.0001	
Colorectal cancer	0 (non‐GERD)	437	Ref.		0 (non‐GERD)	437	Ref.	
	<360 days	50	0.73	(0.54–0.98)	<540 days	67	0.74	(0.57–0.96)
	≥360 days	55	0.73	(0.55–0.97)	≥540 days	38	0.71	(0.51–0.98)
	*p*‐trend		0.007		*p*‐trend		0.006	
Liver cancer	0 (non‐GERD)	295	Ref.		0 (non‐GERD)	295	Ref.	
	<360 days	38	0.83	(0.59–1.17)	<540 days	45	0.75	(0.54–1.02)
	≥360 days	23	0.45	(0.29–0.68)	≥540 days	16	0.43	(0.26–0.72)
	*p*‐trend		0.0002		*p*‐trend		0.0003	
Pancreatic cancer	0 (non‐GERD)	99	Ref.		0 (non‐GERD)	99	Ref.	
	<360 days	7	0.49	(0.23–1.05)	<540 days	9	0.48	(0.24–0.95)
	≥360 days	6	0.37	(0.16–0.85)	≥540 days	4	0.34	(0.13–0.94)
	*p*‐trend		0.005		*p*‐trend		0.0052	

Abbreviations: CI, confidence interval; GERD, gastroesophageal reflux disease; HR, hazard ratio.

^a^ Model adjusted for all covariates, including age, sex, smoking status, alcohol consumption, physical activity, BMI, income, area, and Charlson Comorbidity Index; liver cancer was additionally adjusted for chronic viral hepatitis.

### 3.5 Additional analyses

In additional analyses, we found that GERD patients have a higher number of health checkups (6.33 vs. 5.76) and a higher number of hospital visits per year (31.33 vs. 15.88) compared to the non‐GERD group. We also observed a higher number of thyroid biopsy procedures and polypectomy procedures received among the GERD group (Table S1).

When the number of health checkups and the number of hospital visits per year were included in the full Cox PH model, we observed a significantly increased risk for esophageal, laryngeal, and thyroid cancers. However, no significant result was observed for liver and pancreatic cancers after stratifying by GERD subtypes. Moreover, when adding the thyroid biopsy variable, no significant result was found for thyroid cancer with both GERD subtypes (Table S2).

## 4 DISCUSSION

We examined the association between GERD and several cancer types while controlling for possible confounders, including age, sex, smoking status, alcohol consumption, physical activity, BMI, income, area, and CCI. We found a significant increase in the risk of esophageal cancer (aHR = 3.20 [1.89–5.41]), laryngeal cancer (aHR = 5.42 [2.68–10.96]), and thyroid cancer (aHR = 1.91 [1.55–2.34]) after controlling for all covariates. Our study also observed consistent results when separately examining GERD with esophagitis (K210) and without esophagitis (K219). We also observed stronger effects of GERD in participants with a long duration of GERD treatment (≥360 days or ≥540 days). The increased risk of thyroid cancer among GERD patients could be due to the higher utilization of thyroid biopsy procedures. In contrast, we observed a reduced risk of colorectal, liver, and pancreatic cancers among the GERD group. However, this reduced risk was not consistent when stratified by GERD subtypes (GERD with and without esophagitis) and when controlling for differences in healthcare usage between these two groups.

Previous evidence suggests that repeated exposure to gastric acid in GERD patients can damage and change the esophageal epithelium, leading to esophageal cancer.^8^, ^21^ Epithelial damage can also occur in the larynx and other oral sites when gastric acid refluxes back into those sites, where sites closer to the stomach are more commonly affected. After adjusting for confounding factors, we confirmed a significant association of GERD with laryngeal cancer. The findings for laryngeal cancer were supported by previous studies.^9^, ^10^ A meta‐analysis of 13 case–control studies found an odds ratio (OR) of 1.95 (1.33–2.86) for laryngeal cancers due to GERD.^9^ The other meta‐analysis of 18 case–control studies found 2.47 times increased risk (OR = 2.47 [1.90–3.21]) for laryngeal cancer among GERD patients.^10^ A recent US cohort showed that the increased risk of laryngeal cancer in GERD patients was independent of sex, smoking status, alcohol intake, and follow‐up time.^22^ A previous study using the Korean database also showed a significant increase in laryngeal cancer but with a smaller magnitude (aHR = 2.32 [1.53–3.52]).^16^ However, this study could not assess possible confounders, including smoking, alcohol consumption, and BMI. In addition, we used a more stringent definition for GERD (≥8 weeks of GERD treatment) than previous studies^16^, ^23^, ^24^ (≥1, 2, or 4 weeks of GERD treatment) to increase the accuracy of GERD diagnosis in the claim data.

We observed an increased risk of thyroid cancer in patients with GERD, but the result was attenuated to null after adjusting for having undergone a thyroid biopsy procedure. The increased risk may results from a higher rate of healthcare utilization, leading to a higher screening rate for thyroid cancer among GERD patients. Indeed, we found that GERD patients were more likely to participate in general health checkups and had a higher number of hospital visits per year. A previous study also suggested that people who participate in preventive and health‐seeking behaviors are more likely to screen for cancer than those who do not.^25^ Our study also found that GERD patients had a higher proportion of receiving thyroid biopsy procedures, which are used to screen for thyroid cancer (Table S1). In Korea, although thyroid cancer is not included in the Korean National Cancer Screening Program, it is frequently offered at a low price by health providers and included in the health checkup programs of many Korean hospitals.^26^ In addition, overdiagnosis has been a significant issue in thyroid cancer.^27^, ^28^ Previous studies report the overdiagnosis of thyroid cancer among the Korean population during the time period that match our follow‐up time.^29^, ^30^ The incidence of thyroid cancer increased steeply in Korea, with the age‐standardized incidence rate in both men and women rising from 6.5 per 100,000 in 1999 to 40.2 per 100,000 in 2018, and peaking at 63.4 per 100,000 in 2012. Especially for women, thyroid cancer has become the most common cancer since 2005, while its mortality has slightly decreased.^31^, ^32^

For pancreatic, colorectal, and liver cancers, the observed reduced risks were not consistent among the GERD subgroups. For pancreatic and colorectal cancers, no significant result was observed for GERD without the esophagitis group, and when further controlling for differences in healthcare utilization, the significantly increased risk of pancreatic cancer among GERD with esophagitis was attenuated to null. For liver cancer, no significant result was observed when adjusting for healthcare utilization. In line with our findings, the association between GERD and colorectal and liver cancers in previous studies remains controversial. For colorectal cancer, a cohort study found a significantly increased risk (aHR = 1.76 [1.62–2.90]) among GERD patients; however, the study did not consider other possible confounders including the competing risk of other cancers.^14^ In contrast, another study suggested no increase in the risk of colorectal cancer among patients with Barret's esophagus, a complication of GERD, compared with the general population.^33^ For liver cancer, a meta‐analysis found a significantly increased risk of non‐alcoholic fatty liver disease among GERD patients; however, it is unclear whether this association is causal or due to common risk factors such as obesity.^34^

Liver and colorectal cancers have been included in the Korean National Cancer Screening Program since 2003 and 2004, respectively, allowing the Korean population to access free‐of‐charge or low‐cost screening measures.^35^ For colorectal cancer, previous evidence suggested that screening programs significantly reduce cancer incidence.^36^, ^37^, ^38^ Our study also found that GERD participants were more likely to receive polypectomy procedures (Table S1). For liver cancer, we observed a higher proportion of chronic viral hepatitis (Table 1) but a minor effect of chronic viral hepatitis on liver cancer among GERD participants (Table S3). These findings may suggest the impact of better detection and treatment for precancerous lesions and important risk factors of colorectal and liver cancers, resulting in a decreased risk of these cancers among GERD groups. Unfortunately, our data lack information on cancer screening participation to verify this association. Hence, further studies are needed to examine the effect of GERD on cancer screening participation.

Our study has several strengths, including the use of representative clinical data set for the Korean population. We examined the relationship between GERD and various types of cancer and we were able to control for possible confounding factors that may affect their relationships. In addition, we were able to assess a dose–response relationship by exploring the effect of GERD according to the duration of GERD treatment.

However, several limitations must be considered when interpreting the results of our study. First, GERD may be asymptomatic and thus medication usage may not represent all patients. However, information on clinical symptoms was unavailable in the claims data used in our study. Definition using only diagnosis code (ICD‐10) can lead to overestimation of the GERD due to an upcoding. Therefore, we defined the GERD using diagnosis code combined with prescription code to improve the accuracy of GERD diagnosis. Nevertheless, there is a possibility of underestimation of GERD as patients with gastric problems may be under medication. Second, we lack information on the cancer pathology to examine the effect of GERD on cancer subtypes. Third, we do not have data on participation in cancer screening. In Korea, the national screening programs offer free or low‐cost cancer screening for colorectal, liver, stomach, and breast cancers (lung cancer screening has recently been added since 2019).^35^ This information could be informative when evaluating the risk of these cancers among GERD patients. Fourth, we did not assess laryngeal reflux, which may be more strongly associated with laryngeal cancer if assumed that the action of acid increases the risk of cancer in GERD patients. Laryngopharyngeal reflux is present in patients with GERD, but its development may differ from that of GERD.^39^ However, a recent meta‐analysis found that the risk of laryngeal malignancy is not significantly different between patients with GERD and patients with laryngeal reflux.^10^ Fifth, cancer may take several years from onset to diagnosis, so the current exclusion criteria may not be sufficient to exclude patients who had cancer but were not detected during the diagnosis of GERD.

In conclusion, our study confirms a higher risk of esophageal and laryngeal cancers in patients with GERD after controlling for possible confounding factors. Furthermore, early detection and treatment of precancerous lesions among the GERD group could lead to a lower risk of colorectal, liver, and pancreatic cancers.

## AUTHOR CONTRIBUTIONS

**Chi Lan Tran:** Formal analysis (lead); writing – original draft (lead). **Minji Han:** Data curation (supporting); formal analysis (supporting); methodology (supporting). **Byungmi Kim:** Writing – review and editing (supporting). **Eun Young Park:** Writing – review and editing (supporting). **Young Il Kim:** Writing – review and editing (supporting). **Jin‐Kyoung Oh:** Conceptualization (lead); supervision (lead); writing – review and editing (lead).

## CONFLICT OF INTEREST STATEMENT

The author reports no conflicts of interest in this work.

## ETHICS STATEMENT

Since this study used anonymous secondary data, it was exempt from review by the Institutional Review Board of the National Cancer Center, Korea (NCC2018‐0279).

## Supporting information


Table S1.
Table S2.
Table S3.

## Data Availability

The HEALS dataset which used in this study (NHIS‐2020‐2‐097) is available upon request from the National Health Insurance Sharing Service, https://nhiss.nhis.or.kr/.
