# Supplementary material for: Gastroesophageal reflux disease and risk of cancer: Findings from the Korean National Health Screening Cohort
Source: Cancer Med. 2023 Sep 7;12(18):19163–73. doi: 10.1002/cam4.6500 (PMC10557881; doi:10.1002/cam4.6500)
Supplement: Supplementary file 1 — Table S1. Table S2. Table S3. [file CAM4-12-19163-s001.docx]

**Supplemental Table 1. Healthcare utilization in GERD and non-GERD groups**

| **Characteristics** | **GERD group** (n=10,261) | **Non-GERD group** (n=30,783) | *p*-value |
| --- | --- | --- | --- |
| **Number of health check-up**  *Mean (SD)* | 6.33 (2.62) | 5.76 (3.07) | < .0001‡ |
| **Number of hospital visit per year**  *Mean (SD)* | 31.33 (21.63) | 15.88 (14.35) | < .0001‡ |
| **Receive thyroid biopsy procedure**  *N (%)* | 925 (9.01) | 1,168 (3.79) | < .0001† |
| **Receive polypectomy procedure**  *N (%)* | 2,835 (27.63) | 3,560 (11.56) | < .0001† |

*GERD: gastroesophageal reflux disease; SD: standard deviation.*

*^†^: Chi-squared test.*

*^‡^: two-sample t–test.*

**Supplemental Table 2. Hazard ratios for cancer adjusted for healthcare utilization.**

|  | **GERD** | | | **GERD with esophagitis**  **(K210)** | | | **GERD without esophagitis (K219)** | | |
| --- | --- | --- | --- | --- | --- | --- | --- | --- | --- |
|  | **HR** | **95% CI** | ***p*-value** | **HR** | **95% CI** | **p** | **HR** | **95% CI** | ***p*-value** |
| **Additionally adjusted for health utilization**^†^ | | |  |  |  |  |  |  |  |
| All cancer | 1.00 | (0.93–1.08) | 0.982 | 1.03 | (0.95–1.13) | 0.454 | 0.88 | (0.74–1.05) | 0.150 |
| *Esophageal cancer* | 3.74 | (2.08–6.71) | <.0001 | 3.75 | (1.95–7.21) | <.0001 | 4.36 | (1.14–16.65) | 0.032 |
| *Stomach cancer* | 0.94 | (0.78–1.14) | 0.555 | 0.90 | (0.73–1.11) | 0.328 | 1.14 | (0.74–1.75) | 0.557 |
| *Colorectal cancer* | 0.68 | (0.54–0.85) | 0.0008 | 0.71 | (0.55–0.91) | 0.008 | 0.56 | (0.33–0.93) | 0.026 |
| *Liver cancer* | 0.85 | (0.63–1.15) | 0.284 | 0.94 | (0.68–1.31) | 0.727 | 0.44 | (0.18–1.09) | 0.075 |
| *Pancreatic cancer* | 0.51 | (0.28–0.95) | 0.034 | 0.55 | (0.27–1.12) | 0.098 | 0.43 | (0.12–1.56) | 0.197 |
| *Laryngeal cancer* | 5.01 | (2.36–10.65) | <.0001 | 5.73 | (2.30–14.26) | 0.0002 | 4.60 | (1.12–18.91) | 0.035 |
| *Lung cancer* | 1.01 | (0.80–1.28) | 0.939 | 1.03 | (0.79–1.33) | 0.847 | 0.95 | (0.53–1.71) | 0.861 |
| *Thyroid cancer* | 1.62 | (1.30–2.03) | <.0001 | 1.69 | (1.31–2.19) | <.0001 | 1.43 | (0.89–2.32) | 0.143 |
| **Additionally adjusted for thyroid biopsy procedure**^‡^ | | |  |  |  |  |  |  |  |
| Thyroid cancer | 1.14 | (0.92–1.41) | 0.230 | 1.20 | (0.95–1.53) | 0.133 | 0.99 | (0.64–1.54) | 0.968 |

*GERD: gastroesophageal reflux disease; HR: hazard ratio; CI: confidence interval.*

*^†^ Adjusted for the number of health check-ups and the number of hospital visits per year, in combination with age, sex, smoking status, alcohol consumption, physical activity, BMI, income, and area; liver cancer was additionally adjusted for chronic viral hepatitis B or C.*

*^‡^ Adjusted for receiving thyroid biopsy procedure, in combination with age, sex, smoking status, alcohol consumption, physical activity, BMI, income, and area; liver cancer was additionally adjusted for chronic viral hepatitis B or C.*

**Supplemental Table 3. Liver cancer risk due to chronic viral hepatitis of GERD and non-GERD groups**

|  | **GERD group** (n=10,261) | | **Non-GERD group** (n=30,783) | |
| --- | --- | --- | --- | --- |
| **Chronic viral hepatitis** | HR^†^ (95% CI) | *p*-value | HR^†^ (95% CI) | *p*-value |
| Yes | 3.47 (1.48–8.15) | 0.0043 | 6.81 (4.61–10.08) | <.0001 |
| No | Ref. |  | Ref. |  |

*GERD: gastroesophageal reflux disease; HR: hazard ratio; CI: confidence interval.*

*^†^ Adjusted for age, sex, smoking status, alcohol consumption, physical activity, BMI, income, and area.*
